# Remote ischemic conditioning in intensive care: From bench to bedside

**DOI:** 10.1016/j.aicoj.2025.100018

**Published:** 2026-01-16

**Authors:** Sarah Benghanem, Laure Stiel, Youenn Jouan, Meryl Vedrenne-Cloquet, Yael Levy, Thomas Maldiney, Benjamine Sarton, Hatem Kallel, Nicolas Bréchot, Jérémie Joffre, Romain Gallet, Alexandre Gaudet, Louis Kreitmann

**Affiliations:** a Medical Intensive Care Unit, Cochin Hospital, AP-HP Centre, Paris, France; b University of Paris Cité – Medical School, Paris, France; c Institute of Psychiatry and Neuroscience of Paris (IPNP), INSERM U1266, Paris, France; d Department of Intensive Care Medicine, Groupe Hospitalier de la Région Mulhouse Sud Alsace, Mulhouse, France; e Lipness Team, INSERM Research Team, LNC UMR 1231 and LabEx LipSTIC, University of Burgundy, Dijon, France; f Intensive Care Medicine Department, CHRU Tours, Tours, France; g Cardiovascular Surgical Intensive Care Unit and Cardiac Surgery Department, CHRU Tours, Tours, France; h INSERM, U1100 Center for the Study of Respiratory Diseases, Faculty of Medicine of Tours, Tours, France; i Pediatric Intensive Care Unit, Necker Hospital, AP-HP Centre, Paris, France; j Paediatric Intensive Care Unit, University Hospital Center Félix Guyon, La Reunion, France; k Reunion University, La Reunion, France; l INSERM CIC-EC 1410, La Réunion, France; m Department of Intensive Care Medicine, William Morey General Hospital, Chalon-sur-Saône, France; n Lipness Team, French National Institute of Health and Medical Research (INSERM) Research Center Lipids, Nutrition, Cancer – Joint Research Unit (LNC-UMR) 1231, University of Burgundy, Dijon, France; o General Intensive Care Unit, Purpan Hospital, Toulouse University Hospital, Toulouse, France; p ToNIC Lab (Toulouse NeuroImaging Center), INSERM/UPS UMR 1214, 31300 Toulouse, France; q Intensive Care Unit, French Guiana University Hospital, French Guiana; r Tropical Biome and Immunopathology CNRS UMR-9017, Inserm U 1019, Université de Guyane, French Guiana; s Medical Intensive Care Unit, Georges Pompidou European Hospital, Assistance Publique–Hôpitaux de Paris (AP-HP), Paris, France; t Center for Interdisciplinary Research in Biology, Collège de France, French National Center for Scientific Research (CNRS), French National Institute of Health and Medical Research (INSERM), Université PSL, Paris, France; u Medical Intensive Care Unit, Saint Antoine Hospital, Assistance Publique–Hôpitaux de Paris (AP-HP), Paris, France; v Saint Antoine Research Center, INSERM U938, Sorbonne University, Paris, France; w Cardiology Department, Henri Mondor Hospital, AP-HP, Créteil, France; x Department of Intensive Care Medicine, Critical Care Centre, Lille University Hospital (CHU), 59000, Lille, France; y University of Lille, CNRS, INSERM, Lille University Hospital, Pasteur Institute of Lille, U1019-UMR9017-CIIL – Center for Infection and Immunity of Lille, Lille, France; z Department of Infectious Disease, Faculty of Medicine, Centre for Antimicrobial Optimisation, Imperial College London, London, United Kingdom; A Department of Critical Care Medicine, Imperial College Healthcare NHS Trust, London, United Kingdom

**Keywords:** Remote ischemic conditioning, Intensive care, Stroke, Acute myocardial infarction, Ischemic-reperfusion injury, sepsis, shock, cardiac surgery, cardiac arrest

## Abstract

Ischemia/reperfusion (I/R) injury is a pathological phenomenon involving temporary blood flow restriction (ischemia) followed by a period of reperfusion. This sudden variation induces oxidative stress, inflammation, and mitochondrial dysfunction, leading to severe cellular damage. I/R is a primary driver of organ injury in critically ill patients, particularly in conditions such as myocardial infarction, stroke, cardiac arrest, trauma, cardiac surgery, and various shock states.

Remote ischemic conditioning (RIC), a non-invasive strategy involving repeated controlled episodes of ischemia and reperfusion to a distant organ (typically a limb), has emerged as a potential strategy to attenuate I/R injury through systemic protective mechanisms. RIC can be applied at various time points: i) before ischemia (pre-conditioning); ii) during ischemia (per-conditioning); iii) after reperfusion has started (post-conditioning). While preclinical models have consistently demonstrated its efficacy, clinical trials to date have shown mixed results, with limited impact on key clinical outcomes.

In this narrative review, we first provide a brief historical overview and outline the molecular and cellular mechanisms underlying RIC. Second, we examine current evidence from both animal studies and human trials on the effect of RIC across several conditions such as myocardial infarction, stroke, cardiac arrest, trauma, cardiac surgery and shock. Finally, we discuss ongoing research efforts aimed at optimizing its delivery and identifying patient populations most likely to benefit from its use.

## Introduction

Ischemia/reperfusion (I/R) injury is a pathological process that occurs when blood supply to an organ is transiently interrupted (ischemia) and subsequently restored (reperfusion). First, the interruption of oxygen delivery during the ischemic phase triggers a range of metabolic dysfunctions that can ultimately lead to cell death. Second, reperfusion—which is essential to restore oxygen and nutrient delivery—paradoxically exacerbates cellular damages though oxidative stress, inflammation, and mitochondrial dysfunction [1,2]. This phenomenon is particularly relevant in conditions that are often seen in patients requiring intensive care admission, such as myocardial infarction, stroke, cardiac arrest, trauma, major surgery and shock [3].

In 1986, Murry et al. described in a canine model of myocardial ischemia that four cycles of 5-min of alternating occlusion and reflow of the left anterior descending (LAD) coronary artery applied before 90 min of occlusion and 3 days of reperfusion resulted in a 75% reduction in infarct size [4]. This effect—named ‘ischemic pre-conditioning’—was subsequently reproduced in multiple species. In 1993, Przyklenk et al. extended those findings to demonstrate that intermittent periods of coronary ischemia could protect remote myocardium from subsequent sustained coronary occlusion [5]. They introduced the concept of ‘remote ischemic pre-conditioning’, whereby brief controlled exposure to ischemia followed by reperfusion can prepare tissues located in distant vascular beds to better tolerate a subsequent more prolonged ischemic insult. In 2003, Zhao et al. demonstrated in a canine model that repeated cycles of controlled I/R applied at the start of reperfusion after a prolonged LAD occlusion (i.e., post-conditioning), was as effective as pre-conditioning to reduce infarct size [6]. Subsequently, Kerendi et al. demonstrated in a rat model that remote post-conditioning applied to the renal artery immediately before the onset of coronary artery reperfusion also led to a reduction in infarct size [7], opening new opportunities to protect the myocardium during unpredictable ischemic events.

Translation from preclinical models to human studies was mainly achieved using simple, non-invasive methods such as blood pressure cuff or tourniquet inflation, as landmark studies by Kharbanda et al. [8] and Cheung et al. [9] provided the first clear evidence that such non-invasive techniques can induce remote pre-conditioning in humans. Since these seminal papers, remote ischemic conditioning (RIC) has emerged as a promising, non-invasive and low-risk intervention aimed at reducing I/R injury.

**Fig. 1:**
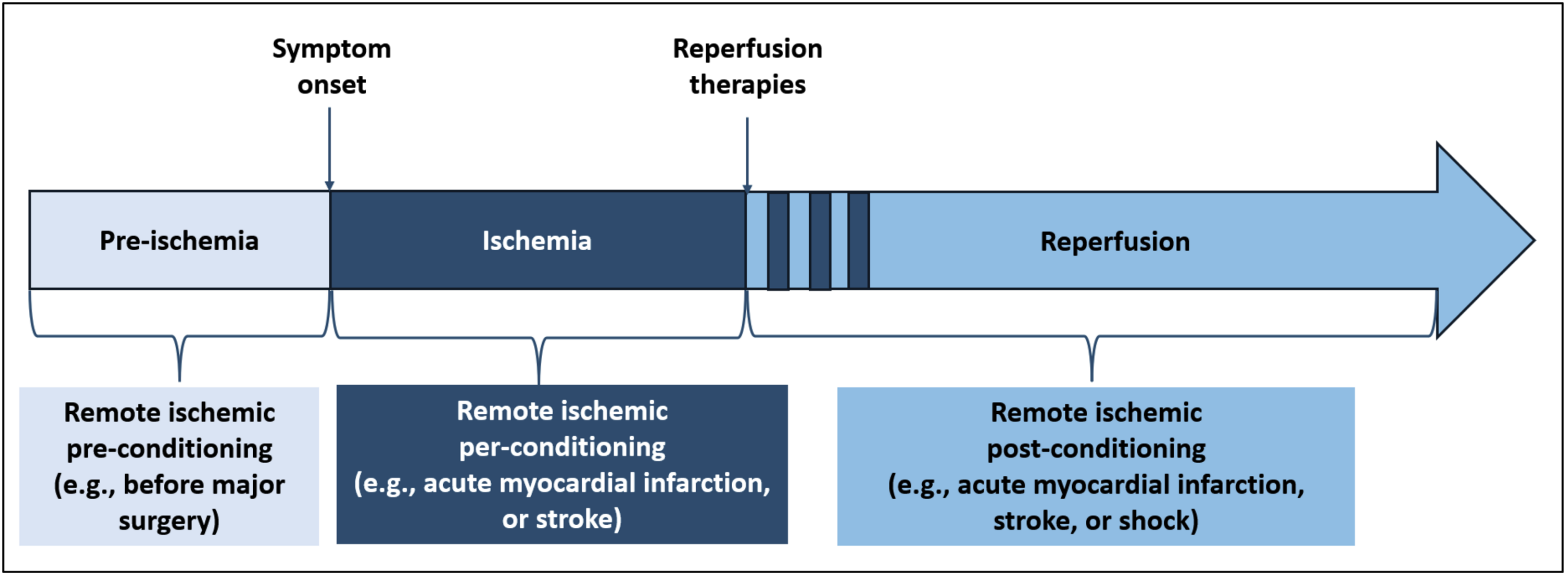
Time points relative to ischemic injury for the use of RIC. RIC: remove ischemic conditioning.

The process of RIC involves the application of short, repeated, reversible, and controlled ischemic and reperfusion episodes in one vascular bed (usually a limb) to trigger the physiological response to ischemia, and thereby protect a vital distant organ (e.g. heart, brain or kidney) from severe and sustained ischemic injury [10]. RIC can be applied at different time points relative to ischemic injury (Fig. 1):

- Pre-conditioning: RIC is applied before an intervention (e.g., elective major surgery);
- Per-conditioning: RIC is initiated during an ongoing ischemic event (e.g. acute myocardial infarction, stroke);
- Post-conditioning: RIC is applied after an ischemic event has occurred and reperfusion has begun (e.g., acute myocardial infarction or stroke, septic or hemorrhagic shock, post-resuscitation shock following cardiac arrest).

While preclinical studies across various animal models have consistently demonstrated protective effects of RIC on organ function, translation to human studies has been inconsistent. This review will detail the molecular and cellular mechanisms of RIC, explore its application in different clinical settings, and discuss the current challenges in translating RIC into the management of patients requiring intensive care unit (ICU) admission.

## Molecular and cellular mechanisms of RIC

**Fig. 2:**
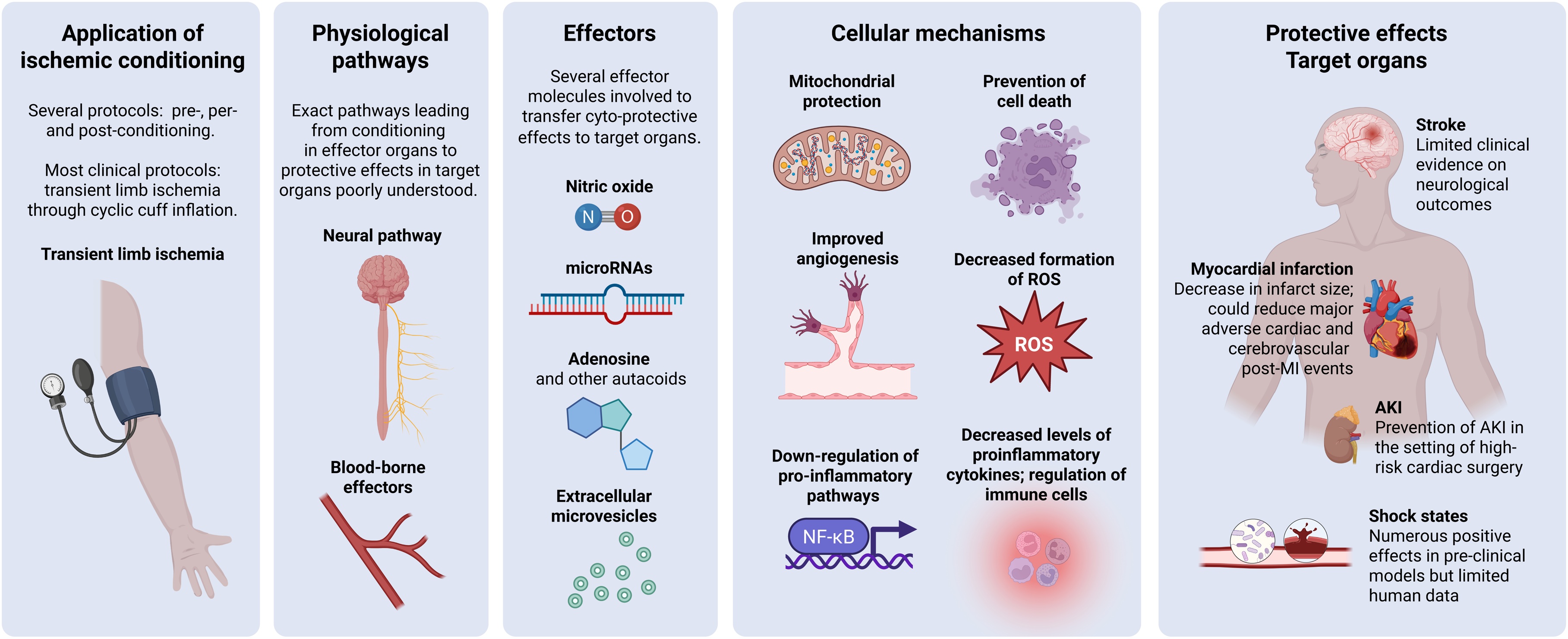
Overview of the pathophysiological mechanisms of remote ischemic conditioning (RIC). AKI: acute kidney injury; MI: myocardial infarction; NF-kB: nuclear factor kappa-light-chain-enhancer of activated B cells; ROS: reactive oxygen species. Created with BioRender.com.

RIC involves a complex biological response that starts at the site of the initial ischemic stimulus (usually a limb) and extends to protect distant organs. Although the precise underlying mechanisms remain unclear, research highlighted some major pathways through which RIC exerts its protective effects [11] (Fig. 2). These include an interplay between specific humoral, neural, and immune-mediated signaling, which activate intracellular downstream pathways supporting cell survival, cellular homeostasis, mitochondrial function, and anti-inflammatory responses [12]. Of note, the molecular mechanisms of RIC may differ according to target organs (e.g., heart vs. brain) and the time of its application (e.g., pre- vs. post-conditioning) [13,14].

By definition, the protective effects of RIC manifest in organs anatomically distinct from the ischemic site, implying that signal transmission must occur through the humoral/systemic circulation or neural pathways. In experimental investigations using parabiosis models (in which two animals share the same circulatory system) or blood transfusion experiments, strong evidence indicates that blood-borne factors play a key role in mediating RIC effects [15]. In addition, the interruption of neural transmission in animal models (via pharmacological blockade or a surgical technique of nerve injury) decreases the cardioprotective effects of RIC [16]. Conversely, the activation of nociceptive fibers at the site of ischemia promotes the release of neurotransmitters, which in turn activates efferent autonomic pathways enhancing protective effects in target organs [17].

The molecular nature of these circulating protective factors remains an area of investigation. However, current evidence suggests that RIC triggers the release of multiple endogenous mediators, such as autacoids (e.g., bradykinin, adenosine, opioids) [18], nitric oxide (NO), extracellular vesicles, microRNAs (e.g.miR-144 [19]) and mitochondria-derived damage-associated molecular patterns (DAMPs). Once these mediators reach target organs, they interact with resident parenchymal cells and attract immune cells to trigger intracellular key survival pathways. These probably include:

- The RISK (Reperfusion Injury Salvage Kinase) pathway, involving the PI3K/Akt (phosphatidyl-inositol-4,5-bisphophate 3-kinase/protein kinase B) and MEK1-ERK1/2 (MAP kinase 1-extracellular signal-regulated kinases) cascades, both contributing to anti-apoptotic and pro-survival signaling [20,21];
- The SAFE (Survivor Activating Factor Enhancement) pathway, including the TNF (Tumor Necrosis Factor) alpha, STAT3 (Signal Transducer and Activator of Transcription 3) and JAK (Janus kinase) cascades, regulating inflammation upon stress conditions [22];
- Protein Kinase C (PKC), a serine/threonine kinase playing a key role in mediating cellular growth [23].

Through these different pathways, RIC ultimately reduces the opening of the mitochondrial permeability transition pore (mPTP), modulates electron transport and activates the ATP-dependent potassium channel, which are necessary to support cell survival [24].

Furthermore, I/R injury promotes a robust inflammatory stress response characterized by the recruitment of innate immune cells, cytokine release and oxidative stress. Thus, RIC appears to mitigate these pro-inflammatory effects through multiple mechanisms:

- Downregulation of the NF-κB (nuclear factor kappa-light-chain-enhancer of activated B cells) and NLRP3 (NOD-, LRR- and pyrin domain-containing protein 3) inflammasome, leading to decreased production of pro-inflammatory cytokines such as interleukin (IL)-1β and IL-18 [25];
- Regulation of the recruitment and infiltration of monocytes, macrophages and T lymphocytes; RIC shifts the pro-inflammatory immune response towards a reparative phenotype, mainly in cerebral ischemia [26];
- Reduction in oxidative stress and the production of reactive oxygen species (ROS): RIC upregulates the production of specific antioxidant defenses such as superoxide dismutase (SOD) and heme oxygenase-1 (HO-1) [27].

In summary, RIC orchestrates a complex protective response that maintains cell survival, attenuates oxidative stress and inflammation, and preserves mitochondrial integrity in target organs. Taken together, these processes contribute to reduce cellular damage, improve ischemic tolerance and accelerate functional recovery upon ischemic conditions.

## Practical delivery of RIC in the clinical setting

In clinical settings, RIC is typically delivered using a standard (usually automated) sphygmomanometer (blood pressure cuff) applied to a limb distant from the organ or tissue at risk. This is most commonly the upper limb (arm), but it can also be applied to the lower limb [28]. A typical RIC protocol involves multiple cycles of brief ischemia and reperfusion (usually 3–5 cycles), with each cycle consisting of 5 min of cuff inflation (ischemia) to a pressure sufficient to occlude arterial blood flow (180−200 mmHg, or 50 mmHg above systolic blood pressure) followed by 5 min of cuff deflation for reperfusion. However, variations in these parameters exist across studies. Specific adverse effects of RIC are mainly mild and include localized pain at the limb where RIC is administered, transient paresthesia, redness or swelling, and skin petechiae. More serious local adverse events such as deep venous thrombosis, permanent nerve injury or arterial dissection exist in theory but have not been reported in large meta-analyses [29,30].

## RIC for cardioprotection in acute myocardial infarction

Clinical translation of RIC has been largely assessed in the setting of ischemic heart disease. Indeed, myocardial infarction is an excellent model of I/R. During the ischemic phase, the occlusion of a coronary artery reduces blood supply to the myocardial tissue, inducing potentially irreversible cellular damage. During the reperfusion phase, the reopening of the artery through interventions such as thrombolysis or angioplasty abruptly restores blood flow. While necessary, this process leads to additional damage caused by reperfusion-related mechanisms. Given that the risk of post-ischemic heart failure mainly correlates with infarct size, cardioprotective therapies like RIC could be of interest. Moreover, the long-term reduction in major adverse cardiac events (MACE) or major adverse cardiac and cerebrovascular events (MACCE) by RIC could represent another important therapeutic avenue.

Although most of the clinical studies used a comparable protocol of RIC (i.e. 3–4 cycles of 5-minute limb ischemia on the upper or lower limb and 5-min reperfusion started before coronary reperfusion) (Table 1), results remain inconsistent. Regarding the effect of RIC on myocardial protection, most of the studies have shown a decrease in infarct size or a relative increase of 20% in myocardial salvage index, this endpoint being assessed using biomarkers or enzyme release, Single Photon Emission Computed Tomography (SPECT) or Magnetic Resonance Imaging (MRI) [[28], [31], [32], [33]]. Importantly, the best results have been observed in patients with larger myocardial infarction (i.e., those with an ischemic time long enough to have a large infarct and potentially respond to cardioprotective therapies, but short enough so that the infarct was not completed) [34]. Conversely, some studies only demonstrated a neutral effect of RIC [[35], [36], [37], [38]]. Several hypotheses could explain these discrepant results. First, the benefit of RIC seems to be increasing over time trough preservation of the left ventricular ejection fraction (LVEF) and only studies with longer follow-up (for example, the CONDI [36] and LIPSIA CONDITIONING trials [37]) have demonstrated a reduction in MACE or MACCE with RIC. Additionally, in the setting of modern treatment of myocardial infarction, it is likely that most of the studies assessing the benefit of RIC on relevant endpoints were underpowered to detect a difference. Thus, several meta-analyses reported a small (∼2%) absolute decrease in acute infarct size, with uncertain or heterogeneous clinical impact [[39], [40], [41]]. Finally, the clinical benefits of RIC could be different among patients with different baseline severity: in the FIRST study, pre-conditioning RIC may decrease the likelihood of MACE at day 90 only in the subgroup of patients with cardiogenic shock or cardiac arrest [42].

**Table 1:** Main randomized controlled trials conducted to assess the effect of remote ischemic conditioning (RIC) in humans with myocardial infarction, acute stroke, cardiac arrest, different shock states and cardiac surgery.

Reference		Patients' characteristics	Intervention	Effects of RIC
**RIC for cardioprotection in myocardial infarction**				
Bøtker et al., *Lancet* (2010) [33]		142 consecutive adult patients with a suspected first acute MI	Per-conditioning:	Increased myocardial salvage.
			4 cycles of 5-min inflation and 5-min deflation of a blood-pressure cuff	
			Started during transport to hospital, and during PCI	
Sloth et al., *Eur Heart J.* (2013), the CONDI trial [36]		333 patients with a suspected first acute STEMI	Per-conditioning:	Reduced major adverse cardiac and cerebrovascular events.
			4 cycles of 5-min inflation followed by 5-min deflation of a blood-pressure cuff (200 mmHg)	
			Started during transport to hospital, and during PCI	
Eitel et al., *Eur Heart J.* (2015), the LIPSIA CONDITIONING trial [47]		696 patients with suspected STEMI and symptoms <12 h undergoing PCI	Per-conditioning: 3 cycles of RIC using inflation for 5 min followed by deflation for 5 min, started before and during PCI	Per- + post-conditioning improves myocardial salvage in comparison to control and to post-conditioning alone.
Stiermaier et al., *Circ Res.* (2019), post-hoc analysis of the LIPSIA CONDITIONING Trial [37]			Post-conditioning: performed after primary PCI via 4 cycles of 30 s balloon occlusions followed by 30 s of reperfusion.	Per- + post-conditioning induces a reduction in MACCE and new congestive heart failure.
Hausenloy et al., *Lancet* (2019), the CONDI-2/ERIC-PPCI trial [38]		5115 patients with suspected STEMI undergoing PCI	Per-conditioning:	No reduction in cardiac death or hospitalization for heart failure at 12 months.
			4 cycles of 5-min inflation and 5-min deflation	
			Started before PCI	
Cheskes et al., *Can J Cardiol.* (2020), the FIRST Study [42]		1667 patients with suspected STEMI undergoing PCI	Per-conditioning: 4 cycles of RIC before PCI	No significant differences in MACE at 30, 60, and 180 days. Patients presenting with cardiogenic shock or cardiac arrest before PCI were less likely to have MACE at 90 days if they received RIC.
**RIC for neuroprotection after stroke**				
Pico et al., *JAMA Neurology* (2022), the RESCUE BRAIN trial [58]		188 patients within 6 h of acute ischemic stroke onset	Per-conditioning:	No significant differences in brain infarction volume growth at 24 h after symptom onset, mortality, or modified Rankin Scale.
			4 cycles of 5-min inflations and 5-min deflations (cuff pressure 110 mmHg above systolic blood pressure), total duration of 40 min	
			Started within 6 h of symptoms onset	
Chen et al., *JAMA* (2022), the RICAMIS trial [60]		1893 patients within 48 h after symptom onset of acute moderate ischemic stroke	Per- + post-conditioning: 5 cycles of cuff inflation (200 mmHg for 5 min) and deflation (for 5 min) bilaterally to the upper limbs twice daily for 10–14 days	Favorable neurological outcome (mRS 0−1) at 90 days was significantly higher in the RIC group.
Blauenfeldt et al., *JAMA* (2023), the RESIST trial [60]		902 patients with prehospital acute ischemic or hemorrhagic stroke < 4 h	Per- + post-conditioning:	No significant improvement of functional outcome at 90 days.
			5 cycles of 5 min of cuff inflation followed by 5 min of cuff deflation (cuff pressure ≤200 mmHg). Treatment started in the ambulance and repeated at least once in the hospital and twice daily for 7 days	
Li et al., *Critical Care* (2024) [63]		80 patients within 24 h of acute ischemic stroke complicating acute myocardial infarction	Per- + post-conditioning:	Decreased incidence in MACCE and improved functional outcomes at 90 days.
			5 cycles of simultaneous bilateral arm ischemia for 5 min + 5 min of reperfusion, during 45 min (cuff pressure 200/60 mmHg)	
			Started within 24 h of stroke onset and twice daily RIC for 2 weeks	
Guo et al., *Stroke* (2025) [62]		547 patients with acute ischemic stroke and intra veinous thrombolysis	Post-conditioning: 4 cycles of 5 min of ischemia (unilateral upper limb, cuff pressure, 200 mmHg) + 5 min of reperfusion, twice daily for 7 days	Favorable neurological outcome (mRS 0−1) at 90 days was not significantly higher in the RIC group.
**RIC after cardiac arrest**				
Bartlett et al., *Resuscitation* (2024) [74]	30 patients with non-traumatic out-of-hospital cardiac arrest		3 cycles of 5-min inflation + 5-min deflation of a blood pressure cuff (200 mmHg)	Safe procedure.
				Secondary endpoints: no difference in mortality or neurological outcome at hospital discharge.
**RIC in shock states**				
Cour et al., *Intensive Care Med.* (2022), the RECO-Sepsis trial [90]	180 patients with septic shock according to the Sepsis-3 definition		Per-conditioning:	No difference in the severity of multiorgan failure as assessed by SOFA score; lower adjusted cumulative day-90 mortality.
			4 cycles of 5-min upper limb ischemia, 5-min reperfusion (cuff pressure 200/0 mmHg)	
			Started within 12 h of onset of septic shock and at 12 and 24 h post-randomization	
Leung et al., *Sci Rep.* (2023) [83]	39 patients with hemorrhagic shock		Per-conditioning:	No difference in clinical endpoints (survival, ICU-free days, ventilator-free days) or plasma levels of cytokines/chemokines.
			4 cycles of 5-min limb ischemia, 5-min of reperfusion (cuff pressure at 250 mmHg)	
			Start within 4 h of shock onset	
**RIC in cardiac surgery**				
Hausenloy et al., *Lancet* (2007) [91]	57 patients undergoing elective coronary artery bypass graft surgery		Pre-conditioning: 3 5-min cycles of right upper limb ischemia, inflated to 200 mmHg, with an intervening 5 min of reperfusion, after induction of anesthesia	Significant reduction in serum troponin-T release at 6, 12, 24, and 48 h after surgery.
Hausenloy et al., *N Engl J Med.* (2015), the ERICCA trial [92]	1612 patients with cardiac surgery		Pre-conditioning:	No reduction in a composite endpoint of death from cardiovascular causes, nonfatal myocardial infarction, coronary revascularization or stroke at 12 months.
			4 cycles of 5-min inflations and deflations of a blood pressure cuff (200 mmHg; if SBP > 185 mmHg, cuff inflated to 15 mmHg above SBP), after induction of anesthesia	
Meybohm et al., *N Engl J Med.* (2015); the RIPHeart Study [93]	1403 patients with cardiac surgery requiring cardiopulmonary bypass		Pre-conditioning: 4 cycles of upper-limb ischemia (5-min blood-pressure cuff inflation to ≥200 mmHg, but at least 15 mmHg higher than the patient’s actual systolic arterial pressure, followed by 5-min cuff deflation) after induction of anesthesia	No reduction in a composite end point of death from any cause, nonfatal myocardial infarction, new stroke, or acute renal failure up to the time of hospital discharge.
Zarbock et al., *JAMA* (2015) [94]	240 patients undergoing high-risk cardiac surgery		Pre-conditioning: 3 cycles of 5-min ischemia, 5-min reperfusion on an upper arm after induction of anesthesia	Reduced rate of AKI and use of renal replacement therapy.
Jia et al., *Circulation* (2024) [95]	509 patients undergoing cardiac surgery with cardiopulmonary bypass.		Pre-conditioning: 4 cycles of 5-min inflation and 5-min deflation on an upper arm 24 h before surgery	Reduced rate of AKI. No difference in perioperative myocardial injury, ICU and hospital LOS, non-fatal myocardial infarction, stroke, and all-cause mortality at day 90.

AKI: acute kidney injury; ICU: intensive care unit; LOS: length-of-stay; MI: myocardial infarction; PCI: percutaneous coronary intervention; SBP: systolic blood pressure; STEMI: myocardial infarction with ST elevation.

However, promising results on RIC have been seriously challenged by the negative findings of the large CONDI-2 trial. This randomized controlled study (RCT) included more than 5000 patients and showed that RIC did not reduce cardiac death or hospitalization for heart failure at 12 months [38]. Moreover, this study included almost all patients with acute myocardial infarction, including old and comorbid patients, distal non-left anterior descending occlusion, short symptoms-to-balloon time or TIMI flow ≥2 at the time of RIC, these different factors being associated with decreased (or suppressed) efficacy of RIC [[43], [44], [45]]. Therefore, studies on a more selected population, albeit much more difficult to complete, are needed.

Last, some data suggest that combining per- with post-conditioning, and to a larger extent, the association of different cardioprotective strategies, may increase the cardioprotective effect of RIC [46,47]. In the LIPSIA CONDITIONING trial, the combination of per- and post-conditioning was superior to standard-of care, although per-conditioning alone was not [47]. In this setting, the results of the CARIOCA trial (NCT03155022) evaluating simultaneous per- and post-conditioning in acute myocardial infarction will probably provide additional information.

## RIC for neuroprotection in stroke and other causes of acute brain injury

Acute ischemic stroke (AIS) is a major cause of I/R brain injury. During recanalization, cerebral reperfusion injury can occur, which manifests as disruption of the blood-brain barrier, cerebral edema, or intracerebral hemorrhage [48]. Although management of AIS has been mainly improved by systemic thrombolysis and endovascular techniques [49,50], a poor neurological outcome at one year is observed in 66% of patients requiring invasive ventilation for acute stroke [51]. Thus, non-pharmacological neuroprotection strategies could be of interest in this situation.

The potential effect of RIC in human AIS was initially suggested by observations of “natural” pre-conditioning; indeed, transient ischemic attacks preceding AIS were associated with a smaller infarct size and fewer clinical deficits in subsequent stroke compared to patients without prior transient events [52,53]. Regarding animal models, some studies conducted in mice indicate that RIC reduces cerebral infarct size [54]. Thus, some recent RCTs have assessed the safety and effectiveness of RIC in humans (Table 1). Hougaard et al. included 443 patients and found that RIC increased tissue survival at 1 month, although there was no difference in clinical outcomes or infarct size [55]. The ReCAST trial involved 26 patients within 24 h of AIS onset, and found lower neurological sequelae at 3 months in the RIC group [56]. The ReCAST-2 study included 60 patients and compared RIC or sham starting within 6 h of stroke onset. A significant difference was observed regarding the level of biomarkers of glial injury (e.g., S100ß protein), with no difference of neuronal injury biomarkers (e.g., neuron-specific enolase—NSE), functional outcome assessed by the modified Rankin Scale score (mRS) or mortality [57]. The RESCUE-BRAIN randomized study compared RIC vs. standard of care in 188 patients within 6 h of AIS: no significant differences on brain infarction volume growth at 24 h after symptom onset, mRS or mortality were observed [58]. The RCT REPOST study found similar results [59]. In the RESIST trial, a multicenter RCT including 902 patients within 4 h of ischemic or hemorrhagic stroke, remote per- and post-conditioning did not significantly improve functional outcome at 90 days [60].

The largest multicenter RCT to date (RICAMIS trial) enrolled 1893 patients with moderate AIS and no intravenous thrombolysis or other endovascular therapies at the acute phase of stroke. This trial found that per- and post- conditioning started in the 48 h after stroke increased the likelihood of an excellent neurological outcome at 90 days [61]. This effectiveness may vary depending on acute-phase therapies administered during the acute phase of ischemic stroke. Indeed, the SERIC IVT study [62] tested the efficacy of remote post-conditioning in patients who underwent thrombolysis and found no significant difference in excellent neurological outcomes at 90 days. Finally, the effect of RIC in the dual cardiac and neurological indication has been poorly evaluated; Li et al. assessed the effect of RIC among patients with AIS following an acute myocardial infarction [63]. In this RCT including 80 patients, RIC improved functional outcomes at 3 months and decreased the incidence of MACCE at day 90. Importantly, all these studies were conducted in stroke units and not in ICUs; thus, the effect of RIC in the most critically ill patients with AIS remains to be assessed.

The effect of RIC in other causes of acute brain injury has also been explored; however available data are limited in comparison to stroke. In traumatic brain injury, a non-randomized interventional study on 40 patients found that RIC performed within 1 h of admission was associated with lower levels of biomarkers of glial and neuronal injury (S-100B protein, and NSE, respectively) at 6 and 24 h post-trauma, with no difference in clinical outcomes [64]. In subarachnoid hemorrhage, a retrospective case-control study on 82 patients found that RIC increased the likelihood of a good neurological outcome at discharge [65]. A single-center RCT confirmed this result, also suggesting that the improvement of neurological outcome could be mediated by a lower incidence of secondary vasospasm [66].

## RIC for neuroprotection after cardiac arrest

Cardiac arrest (CA) is a relatively pure model of I/R injury, inducing a state of global body ischemia and reperfusion that can result in end-organ damage. Before the return of spontaneous circulation (ROSC), the complete cessation (no flow) or limitation (low flow) of cerebral blood flow can lead to hypoxic ischemic brain injury (HIBI) and poor neurological outcome [67]. Indeed, around two thirds of deaths after CA are related to severe and irreversible HIBI, highlighting the need for neuroprotective therapies [67].

HIBI pathophysiology includes global cerebral hypoxic ischemia, excitotoxicity, metabolic acidosis, intracellular calcium overload, mitochondrial injury and inflammation [68]. Restoration of blood flow after CA is also associated with inflammation that may cause neurons and glial cells injury [68]. Thus, RIC has pluripotent effects that could reduce the detrimental effect associated with I/R after CA, in the heart (as described previously) but also and above all in the brain [69] (Table 1).

Regarding animal models, remote ischemic per-conditioning (i.e., during cardiopulmonary resuscitation—CPR) in porcine models improved the left ventricular ejection fraction (LVEF) at 1 and 4 h, as well as neurological outcome at 24 and 48 h [70,71]. Nevertheless, in this animal model, RIC was conducted using 4 controlled 20-s pauses during the first 3 min of CPR, which is not currently recommended in humans. Another animal study found that RIC reduced cardiac biomarkers, with a trend toward improved neurological outcomes [72]. RIC also seems to inhibit hippocampal neuronal apoptosis and mitophagy in rats, suggesting neuroprotective effects [73].

In humans, only one pilot randomized study assessed the safety of RIC in 30 patients with out-of-hospital CA (OHCA). Although RIC was a safe procedure, there was no difference regarding mortality or neurological outcome at hospital discharge [74]. Therefore, the potential clinical benefit of RIC in the context of cardiac arrest remains to be determined. Ongoing trials, including the multicenter RECO-OHCA RCT (NCT06306625) and the monocentric RIPOST-CA RCT (NCT06473207), are anticipated to provide further insights into this question.

## RIC in different shock states

Hemorrhagic shock and its subsequent resuscitation induce a state of global body ischemia and reperfusion that can result in end-organ damage and eventually death. Several pre-clinical studies have assessed the effect of RIC to amend the extent of organ failures in experimental hemorrhagic shock (Table 2) [[75], [76], [77], [78], [79], [80], [81], [82]]. Similar protocols were used to induce hemorrhagic shock in anesthetized mice [78,82], rats [[75], [76], [77],^79^,^80^] or pigs [81] by withdrawing and then returning blood (35–50 % of estimated total blood volume) to induce and maintain hypotension, with a mean arterial pressure of approximately 30−45 mmHg for 30−60 min. RIC was mainly induced through limb or mesenteric ischemia [75]. These studies have reported a positive effect of RIC on a variety of endpoints, including survival [77,79,80], neurological outcomes [77], myocardial function, hemodynamic status [79,80], lung physiology and histology [75,76,78], liver damage [78,82], end-organ and systemic inflammation [75,76,78,79,82].

**Table 2:** Pre-clinical and human studies of remote ischemic conditioning (RIC) in the setting of hemorrhagic shock.

Reference	Species/animal model	Intervention	Main effects of conditioning
Tamion et al., *Am J Physiol Gastrointest Liver Physiol.* (2002) [75]	Rat (n = 80)	Intestinal pre-conditioning: 4 cycles of 1-min mesenteric artery occlusion + 10-min reperfusion	Increased mesenteric blood flow, decreased intestinal lactate accumulation, decrease production of TNF-alpha, decreased fluid requirements and lung edema.
Jan et al., *Resuscitation* (2011) [76]	Rat (n = 36)	Pre-conditioning: 3 cycles of 10-min limb ischemia + 10-min reperfusion	Improved gas exchange, lung histology, decreased lung edema, decreased inflammatory cytokine levels in BAL.
Hu et al., *Shock* (2014) [77]	Rat (n = 21)	Pre-conditioning: 4 cycles of 5-min limb ischemia + 5-min reperfusion	Higher survival, better neurological function, improved myocardial performance and sublingual microvascular flow index.
Leung et al., *Ann Surg.* (2015) [78]	Mouse	Pre-, per- and post-conditioning: 4 cycles of 5-min limb ischemia + 5-min reperfusion	Improved lung and hepatic histology, decreased levels of transaminases, decreased systemic inflammation (TNF-alpha).
Huang et al., *Shock* (2018) [79]	Rat (n = 50)	Per- and post-conditioning: 4 cycles of 5-min limb ischemia + 5-min reperfusion	Improved survival, improved myocardial function (decreased levels of troponin, increase ejection fraction), decreased systemic inflammation.
Dai et al., *Cardiovasc Drugs Ther.* (2019) [80]	Rat	Pre-conditioning: 4 cycles of 5-min limb ischemia + 5-min reperfusion	Higher survival. Higher blood pressure and preload indices, lower urea levels.
Shaylor et al., *Shock* (2020) [81]	Pig (n = 16)	Pre-conditioning: 4 cycles of 5-min limb ischemia + 5-min reperfusion	No difference in survival.
Naraiah Mukkala et al., *Mitochondrion* (2023) [82]	Mouse	Pre-conditioning: 4 cycles of 5-min limb ischemia + 5-min reperfusion	Improved hepatic histology and decreased levels of transaminases.
Leung et al., *Sci Rep.* (2023) [83]	Human (n = 50)	Pre- and post-conditioning: 4 cycles of 5-min limb ischemia + 5-min reperfusion	No difference in clinical endpoints (survival, ICU-free days, ventilator-free days) or plasma levels of cytokines/chemokines.

BAL: bronchoalveolar lavage; LVEF: left ventricular ejection fraction; TNF: tumor necrosis factor.

To our knowledge, only one RCT has been conducted to investigate the effect on RIC in human hemorrhagic shock [83] (Table 1). In this single-center RCT, 50 patients were randomized to RIC vs. sham within 4 h of onset of hemorrhagic shock; there was no difference in any clinical endpoint, including 28-day mortality. There was also no difference in neutrophil oxidative burst activity, expression of adhesion molecules, and on plasmatic levels of myeloperoxidase, IL-6, IL-10 and TNF-alpha. This negative result could be due to the small sample size; moreover, the median time of application of RIC was at 2 h post-injury, likely representing a post-resuscitative phase of injury where RIC is thought to have a lower protective effect.

RIC has been also investigated in experimental septic shock (Table 3) [[84], [85], [86], [87], [88]], where sepsis was induced most often by intraperitoneal [85,87,88] or intravenous [84] injection of lipopolysaccharide (LPS) in mice [85,87,88], rats [84] or sheep [86]. RIC was induced through limb [85,87,88], mesenteric [84] or aortic [86] ischemia at the onset of sepsis in all studies, and maintained at several time points following sepsis onset in some [85,87,88]. Overall, these studies have demonstrated a positive impact of RIC on survival [85,85,86,87,88], cardiovascular status [86,88], microcirculation [86], end-organ damage (i.e., lung, kidney, liver) [84,86,88] and plasmatic levels of pro- and anti-inflammatory cytokines [84,85].

**Table 3:** Pre-clinical models of remote ischemic conditioning (RIC) in the setting of septic shock.

Reference	Species/animal model	Intervention	Main effects of conditioning
Tamion et al, *Am J Physiol Gastrointest Liver Physiol.* (2007) [84]	Rat, intravenous injection of LPS	Intestinal pre-conditioning: 4 cycles of 1-min mesenteric artery occlusion + 4-min reperfusion	Decreased fluid requirements, lung edema, intestinal lactate production and expression of proinflammatory cytokines, intestinal injury.
Kim et al., *J Inflamm.* (2014) [85]	Mouse, intraperitoneal injection of LPS	Per- and post-conditioning: 3 cycles of 10-min limb ischemia + 10 min reperfusion	Increased survival, decreased plasma levels of proinflammatory cytokines, increased levels of anti-inflammatory cytokines.
Orbegozo Cortes et al., *Shock* (2016) [86]	Sheep (n = 14), injection of autologous feces into abdominal cavity	Per- and post-conditioning (at sepsis induction and 4-hourly until the 30^th^ hour): 4 cycles of 2-min aortic clamping + 4-min reperfusion	Increased survival, improved hemodynamics, renal function and sublingual microcirculation; decreased troponin; increased PaO_2_/FiO_2_ ratio.
Joseph et al, *J Surg Res.* (2017) [87]	Mouse (n = 44), intraperitoneal injection of LPS	Per- and post-conditioning (at 0, 2, 6 h post-sepsis induction): 6 cycles of 4-min limb ischemia + 4-min reperfusion	Increased survival.
Honda et al., *Basic Res Cardiol.* (2019) [88]	Mouse, intraperitoneal injection of LPS	Per-conditioning at sepsis induction: 4 cycles of 5-min limb ischemia + 5-min reperfusion	Increased survival, improved cardiac function (decreased troponin, increased cardiac output), decreased liver injury, improved kidney function.

LPS: lipopolysaccharide; PaO_2_/FiO_2_: arterial partial pressure in O_2_ over inspired fraction of O_2_.

Regarding human data, the prospective single-arm trial of Kiudulaite et al. included 26 patients with sepsis; this study suggested that RIC may improve sublingual microcirculatory flow during the first 12 h after the intervention [89]. RECO-Sepsis was a multicentric RCT including 180 septic shock patients, randomized to RIC or a sham procedure within 12 h after the onset of shock [90]. Cumulative mortality at day 90 was 27.6% in the interventional group and 39.6% in the control group. This result was not statistically different in univariate analysis, but significant after adjustment on pre-specified variables, with an adjusted hazard ratio of 0.59 (95%CI 0.35–0.99; p = 0.049) (Table 1).

In conclusion, RIC appears to have a wide-ranging positive impact on organ function and systemic inflammation in preclinical models of hemorrhagic and septic shock, but clinical translation has not been convincingly demonstrated so far. Several reasons for this can be postulated: limited sample size of human RCTs; higher heterogeneity in human subjects compared to animal models. Finally, remote ischemic post-conditioning may be challenging to deliver in clinical situations where the onset of shock is often impossible to anticipate and, in some cases, even difficult to determine precisely (e.g., in septic shock).

## RIC in the setting of cardiac surgery

As discussed above, pre-clinical models have shown that post-conditioning was as effective as pre-conditioning, but in the clinical setting, pre-conditioning remains the modality of choice. Since pre-conditioning is impossible to deliver in the context of acute diseases of unpredictable onset, efforts to demonstrate clinically meaningful effects of pre-conditioning in critically ill patients have focused on major surgery. Cardiac surgery, associated with I/R injury irrespective of whether it involves cardiopulmonary bypass or not, has been an important focus of investigation (Table 1).

Regarding human data, the first RCT conduced by Hausenloy et al. included 57 patients undergoing elective coronary artery bypass graft (CABG) surgery; this study showed a significant reduction in serum troponin T release at 6−48 h after surgery in the RIC group, compared to control procedure [91]. The ERICCA trial was a multicenter RCT where 1612 patients undergoing on-pump CABG surgery (with or without valve surgery) were randomized to pre-conditioning vs. sham, with no significant effect on a composite primary endpoint (death from cardiovascular causes, nonfatal myocardial infarction, coronary revascularization, or stroke at 12 months) [92]. The RIPHeart trial randomized 1403 patients undergoing cardiac surgery to a similar pre-conditioning protocol vs. sham and also found no difference in major clinical outcomes [93].

Conversely, several trials have documented a positive impact of RIC on the incidence of acute kidney injury (AKI) after cardiac surgery. The Renal-RIPC trial enrolled 240 patients undergoing high-risk cardiac surgery; compared to controls, patients undergoing pre-conditioning had a lower incidence of AKI, use of renal replacement therapy and ICU length-of-stay [94]. Similar findings were obtained in a recent single-center RCT on 509 patients undergoing elective cardiac surgery requiring cardiopulmonary bypass, with a significant lower incidence of AKI in the pre-conditioning group [95]. Several meta-analyses confirmed these results [96,97]. Thus, pre-conditioning in cardiac surgery seems to be a promising protective measure regarding AKI.

Interestingly, in the ERICCA and RIPHeart trials (both negative), the majority of patients received propofol during anesthesia, while in the Renal-RIPC trial (with positive results), this drug was not used [98]. As propofol and volatile anesthetics have been shown to diminish or even abrogate the cardioprotective effects of RIC [[99], [100], [101]], a different exposure to these drugs could explain the conflicting results observed across studies on the effect of RIC in the setting of cardiac surgery.

## Further research

Despite promising preclinical evidence, the translation of RIC into clinical practice remains inconsistent across a variety of pathologies. Several key areas warrant further investigation to better define the role of RIC in clinical practice. First, a deeper and more comprehensive understanding of the molecular and cellular pathways involved in RIC is essential. While humoral and neural pathways have been identified as major mediators, the exact molecular nature of the circulating protective factors remains elusive. Advances in proteomics, metabolomics, and transcriptomics may help uncover novel biomarkers and signaling pathways that could enhance the efficacy and reproducibility of RIC. Second, the majority of studies have evaluated the effect of RIC using external devices; yet, endogenous or natural ischemic conditioning is likely common in critically ill patients. For example, Stevic et al. demonstrated that spontaneous ventricular fibrillation in ST-Segment Elevation Myocardial Infarction (STEMI) patients may limit myocardial infarction size at the acute stage, without significant difference in infarct scar size [102]. Further data are needed to evaluate the effect of endogenous ischemic conditioning. Third, the timing, duration, and frequency of RIC using external devices require standardization. Preclinical studies suggest that the effectiveness of RIC may vary depending on when (i.e., pre-, per-, or post-conditioning) and how (i.e., number and duration of ischemic cycles, optimal limb segment to maximize RIC effect) it is applied. More clinical trials are needed to establish which RIC protocols offer the best clinical outcomes, and assess the potential interest of combinations of RIC delivery (pre- and post-, or per- and post-conditioning, for example). Fourth, future RCTs should consider individualized approaches to account for patient heterogeneity. For instance, large-scale trials such as CONDI-2 in myocardial infarction failed to demonstrate significant benefits, possibly due to the inclusion of diverse patient populations with varying levels of comorbidities, infarct sizes, and ischemic durations. Future studies should also focus on identifying specific subgroups that would benefit most from RIC. These subgroups could include younger patients, patients with longer ischemic times, and patients undergoing specific interventions (e.g., cardiac surgery or mechanical thrombectomy for stroke). Finally, integration of RIC procedures into a multimodal therapeutic management bundle (for example, in combination with other neuroprotective strategies such as therapeutic hypothermia after cardiac arrest) could also be of interest.

In summary, large, well-powered, and rigorously designed RCTs are essential to demonstrate the clinical effects of RIC. While meta-analyses suggest potential benefits [30,[39], [40], [41]], most individual trials remain underpowered or lack long-term follow-up. In myocardial infarction, the sub-Saharan African multicenter RCT RIC-AFRICA will include 1200 adult patients with STEMI and no access to percutaneous coronary intervention; this trial will assess the impact of RIC on the composite endpoint of 30-day mortality and heart failure [103]. In OHCA, the French multicenter RECO-OHCA (NCT06306625) trial will enroll 220 patients to investigate whether the effect of RIC (started as soon as possible after inclusion and repeated at 12 and 24 h) can decrease the incidence of a composite outcome of death, multiorgan failure and/or severe neurological injury. The RIPOST-CA study (NCT06473207) will also assess the effect of RIC at 4, 12 h and 24 h in OHCA patients. Finally, the RIPOST-Sepsis trial, a multicenter RCT will further investigate the effect of RIC in the context of septic shock.

## Conclusion

Remote ischemic conditioning (RIC) represents a promising, simple, low-cost and non-invasive strategy to mitigate ischemia/reperfusion injury, a key driver of organ dysfunction in critically ill patients. While robust preclinical data support its protective effects across multiple organ systems, clinical trials in populations such as myocardial infarction, stroke, traumatic brain injury, cardiac arrest, cardiac surgery, and various shock states have yielded inconsistent results. Ongoing and futures clinical trials will be crucial to clarify the potential therapeutic effects of RIC, optimize its timing and delivery protocols, and identify subsets of critically ill patients who may derive the greatest benefit. Rigorous translational efforts will be critical to move RIC from bench to bedside in the intensive care setting.

## CRediT authorship contribution statement

SB, LK: conceptualization, data curation, writing of original draft, review and editing, investigation, illustrations, supervision and project administration. LS, YJ, RG, LK: data curation, writing of original draft, review and editing, investigation. All other authors: writing, review and editing.

## Consent for publication

Not applicable.

## Ethics approval and consent to participate

Not applicable.

## Declaration of Generative AI and AI-assisted technologies in the writing process

During the preparation of this work, the authors used ChatGPT 5 for grammar and syntax checking. After using this tool, the authors reviewed and edited the content as needed and take full responsibility for the content of the published article.

## Funding

No specific funding for this study.

## Availability of data and material

Not applicable.

## Declaration of competing interest

All authors declare that they have no conflict of interest related to this work.
